# Simultaneous PET/fMRI Detects Distinctive Alterations in Functional Connectivity and Glucose Metabolism of Precuneus Subregions in Alzheimer’s Disease

**DOI:** 10.3389/fnagi.2021.737002

**Published:** 2021-09-24

**Authors:** Miao Zhang, Wanqing Sun, Ziyun Guan, Jialin Hu, Binyin Li, Guanyu Ye, Hongping Meng, Xinyun Huang, Xiaozhu Lin, Jin Wang, Jun Liu, Biao Li, Yaoyu Zhang, Yao Li

**Affiliations:** ^1^Department of Nuclear Medicine, Ruijin Hospital, Shanghai Jiao Tong University School of Medicine, Shanghai, China; ^2^School of Biomedical Engineering, Shanghai Jiao Tong University, Shanghai, China; ^3^Department of Neurology & Institute of Neurology, Ruijin Hospital, Shanghai Jiao Tong University School of Medicine, Shanghai, China; ^4^Collaborative Innovation Center for Molecular Imaging of Precision Medicine, Ruijin Center, Shanghai, China

**Keywords:** Alzheimer’s disease, mild cognitive impairment, hybrid PET/MRI, glucose metabolism, functional connectivity, precuneus, subregions

## Abstract

As a central hub in the interconnected brain network, the precuneus has been reported showing disrupted functional connectivity and hypometabolism in Alzheimer’s disease (AD). However, as a highly heterogeneous cortical structure, little is known whether individual subregion of the precuneus is uniformly or differentially involved in the progression of AD. To this end, using a hybrid PET/fMRI technique, we compared resting-state functional connectivity strength (FCS) and glucose metabolism in dorsal anterior (DA_pcu), dorsal posterior (DP_pcu) and ventral (V_pcu) subregions of the precuneus among 20 AD patients, 23 mild cognitive impairment (MCI) patients, and 27 matched cognitively normal (CN) subjects. The sub-parcellation of precuneus was performed using a K-means clustering algorithm based on its intra-regional functional connectivity. For the whole precuneus, decreased FCS (*p* = 0.047) and glucose hypometabolism (*p* = 0.006) were observed in AD patients compared to CN subjects. For the subregions of the precuneus, decreased FCS was found in DP_pcu of AD patients compared to MCI patients (*p* = 0.011) and in V_pcu for both MCI (*p* = 0.006) and AD (*p* = 0.008) patients compared to CN subjects. Reduced glucose metabolism was found in DP_pcu of AD patients compared to CN subjects (*p* = 0.038) and in V_pcu of AD patients compared to both MCI patients (*p* = 0.045) and CN subjects (*p* < 0.001). For both FCS and glucose metabolism, DA_pcu remained relatively unaffected by AD. Moreover, only in V_pcu, disruptions in FCS (*r* = 0.498, *p* = 0.042) and hypometabolism (*r* = 0.566, *p* = 0.018) were significantly correlated with the cognitive decline of AD patients. Our results demonstrated a distinctively disrupted functional and metabolic pattern from ventral to dorsal precuneus affected by AD, with V_pcu and DA_pcu being the most vulnerable and conservative subregion, respectively. Findings of this study extend our knowledge on the differential roles of precuneus subregions in AD.

## Introduction

The precuneus plays a critical role in fundamental cognitive functions including self-processing, memory, visual-spatial imagery, etc. (Cavanna and Trimble, 2006). In addition, it has been identified as a cortical hub for integrative processing of segregated systems in the brain (Buckner et al., 2009; Tomasi and Volkow, 2011). Likely due to its continuous high baseline activity and/or metabolism (Shokri-Kojori et al., 2019), the precuneus is susceptible to amyloid β (Aβ) deposition (Buckner et al., 2009), a pathophysiological biomarker of Alzheimer’s Disease (AD). Increasing evidence has shown that the precuneus suffered disrupted functional connectivity (FC; Greicius et al., 2004; Damoiseaux et al., 2012) and glucose hypometabolism (Kapogiannis and Mattson, 2011; Pascoal et al., 2019), which might have a significant impact on the network degeneration of AD (Drzezga et al., 2011; Drzezga, 2018).

Despite its important role in the pathogenesis of AD as a whole cortical structure, the precuneus has been recognized as an anatomically and functionally heterogeneous brain region. Based on its cytoarchitecture as well as anatomical and functional connectivities, the precuneus has been broadly subdivided, or hieratically classified, into three clusters, namely the dorsal-anterior, dorsal-posterior, and ventral/central subregions (Margulies et al., 2009; Zhang and Li, 2012; Wang et al., 2019; Luo et al., 2020; Ye et al., 2021). Among them, the dorsal anterior and posterior subregions are majorly involved in sensorimotor and visual-related functions, and the ventral/central subregion mostly participates in higher-order cognitive and self-related functions (Cauda et al., 2010). Previous studies have demonstrated selective vulnerability of these subregions affected by AD. Specifically, while the ventral/central subregion of the precuneus showed significantly reduced resting-state FC with other brain regions in AD patients, the dorsal subregion was unchanged or slightly disturbed (Xia et al., 2014; Wu et al., 2016; Khan et al., 2020). Nevertheless, how the internal functional integrity of each subregion is affected by AD remains to be elucidated.

Besides functional disruptions, glucose metabolism, as a surrogate for neuronal activity, has been shown to reduce in the precuneus and suggested to predict the progression of AD (Kato et al., 2016; Mutlu et al., 2017). Previous studies further suggested that the disruptions in neuronal activity and FC are causally linked and may both be consequences of neurotoxic amyloid aggregation (Drzezga et al., 2011; Marchitelli et al., 2018). However, whether the glucose metabolism of each subregion is uniformly or differentially involved in the progression of AD and their associations with the functional disruption are not well understood.

The aim of this study is to investigate the functional and metabolic activities in the subregions of the precuneus across different stages over the spectrum of AD. A hybrid PET/fMRI technique was employed to measure the FC strength (FCS) with the blood oxygen-level dependent (BOLD) signal (Liang et al., 2013) and glucose metabolism with fluorine-18 (^18^F) fluorodeoxyglucose (FDG) in AD patients, mild cognitive impairment (MCI) patients and age- and education-matched cognitively normal (CN) subjects. The functional and metabolic signals are known to change with head motions, different physiological states or moods of the participants (Waites et al., 2005), which are particularly susceptible to patients with MCI and AD in sequential and long acquisitions. The use of hybrid PET/MRI technique minimizes these fluctuations in a time-efficient manner, provides better temporal and spatial registrations between the two modalities, and enables a more accurate evaluation of the interrelated functional and metabolic changes due to AD (Cecchin et al., 2017). In addition, an FC-based K-means clustering algorithm (Ye et al., 2021) was adopted to subdivide the precuneus into the dorsal-anterior, dorsal-posterior and ventral subregions. Results of this study may pave the way for further understanding of selective disruptions in subregions of the precuneus in AD.

## Materials and Methods

### Study Design

A total of 70 participants were included in the study [27 CN: 15 females and 12 males, mean age: 67.48 years (range: 52–83 years); 23 mild cognitive impairment (MCI) patients: 15 females and eight males, mean age: 70.56 years (range: 49–82 years); and 20 AD patients: 15 females and five males, mean age: 66.00 years (range: 46–83 years)]. Demographics of each group are listed in Table 1. The participants were recruited from ongoing studies of aging at Memory Clinic of Ruijin Hospital, Shanghai, China. All participants underwent cognitive assessments with the clinical dementia rate (CDR) scale. Those with CDR = 0.5 and CDR ≥ 1 were clinically diagnosed as MCI and AD, respectively. All subjects in the CN group had CDR = 0. Participants also completed the Mini-Mental State Examination (MMSE) as part of their evaluations. Exclusion criteria included: (1) psychiatric or other neurological diseases; (2) pregnancy or renal failure (critical for PET imaging); (3) major systemic disease; (4) history of traumatic brain injury; and (5) drug or alcohol addiction. All participants or their designees provided written informed consent to the study as part of the Institutional Review Board-approved protocol by Ruijin Hospital, which is in accordance with the Helsinki Declaration and its later revised ethical standards.

**Table 1:** Demographic and clinical information.

	CN	MCI	AD	p-value
N	27	23	20	
Gender (F/M)	15/12	15/8	15/5	0.386
Age, year	67.48 (7.44)	70.56 (8.30)	66.00 (9.44)	0.188
Education, year	13.52 (2.82)	12.78 (3.72)	11.65 (3.70)	0.183
MMSE	29.37 (0.79)	27.26 (1.86)^a^	21.50 (4.65)^b,c^	<0.001
CDR 0/0.5/≥1	0	0.5	≥1	

*Values are mean (SD). Chi-square was used for gender comparisons, one-way ANOVA with post hoc Bonferroni comparisons was used for comparisons of age, education and MMSE scores among all groups. ^a^CN ≢ MCI (*p* < 0.05); ^b^MCI ≢ AD (*p* < 0.05); ^c^CN ≢ AD (*p* < 0.05). Abbreviations: CN, cognitively normal; MCI, mild cognitive impairment; AD, Alzheimer’s Disease; MMSE, Mini-Mental State Examination; CDR, clinical dementia rating*.

### Image Acquisitions

All imaging data were acquired on a Biograph mMR scanner (Siemens Healthcare, Erlangen, Germany) in a single session. Each subject was required to fast for at least 6 h before receiving a bolus injection of the ^18^F-FDG using a mean dose of 207.8 ± 35.5 MBq (range 140.6–329.3 MBq). Simultaneous PET/MR images were obtained at 40–60 min post injection. Structural MR images were acquired using T1-weighted magnetization-prepared rapid gradient echo (MPRAGE) sequence: repetition time (TR) = 1,900 ms, echo time (TE) = 2.44 ms, flip angle (FA) = 9°, field of view (FOV) = 256 × 256 mm^2^, voxel size = 0.5 × 0.5 × 1.0 mm^3^, number of slices = 192. Resting-state functional MRI (fMRI) images were acquired using a gradient-echo echo planar imaging sequence: TR = 2,000 ms, TE = 22 ms, FA = 90°, FOV = 192 × 192 mm^2^, voxel size = 3.0 × 3.0 × 3.0 mm^3^, spacing between slices = 3.75 mm, number of slices = 36, number of volumes = 240. During the scan, the participants were instructed to lay supine with eyes closed but remain awake and avoid systematic thinking. The PET images were acquired in sinogram mode for 15 min. A matrix size of 344 × 344 × 127 was used, resulting in a voxel size of 2.1 × 2.1 × 2.0 mm^3^. In order to improve the accuracy of the standardized uptake value, we applied an additional model-based bone compartment on the basis of Dixon method for attenuation correction. After corrections of random coincidences, dead-time, scatter and photon attenuation, the PET images were reconstructed using ordered subset expectation maximization algorithm (four iterations, 21 subsets). Post-filtering was performed using a 2 mm full width half-maximum (FWHM) Gaussian filter.

### PET Data Processing

The PET data were processed using the statistical parametric mapping software (SPM12). All PET images of each subject were registered to their own T1-weighted images using affine transformation and corrected for partial volume effects using the Müller-Gärtner method (Müller-Gärtner et al., 1992) in the PETPVE12 toolbox (Gonzalez-Escamilla et al., 2017). The T1-weighted images were spatially normalized to the Montreal Neurological Institute (MNI)-152 template and the transformation parameters were subsequently applied to warp the PET images to the MNI space. Afterwards, the PET data were quantified using the standard uptake value ratio (SUVR) referenced by the mean uptake in the cerebellar gray matter and smoothed with a 6 mm FWHM Gaussian kernel.

### fMRI Data Preprocessing

All fMRI images were processed using the Analysis of Functional Neuroimaging (AFNI 21.0.06) software (Cox, 1996). The first four volumes of each subject were discarded to avoid noises due to instable adaption to the scanning. The remaining volumes underwent slice timing and head motion corrections. All subjects had a mean framewise displacement ≤0.5 mm and a percentage of missing data points ≤40%. Nonlinear spatial normalization to MNI space were applied to all the images. Then, temporal band-pass filtering with bandwidth of 0.01–0.1 Hz was performed to reduce the effect of low-frequency drifts and high-frequency noises. Several nuisance signals, including estimated motion parameters, their first derivatives, as well as the averaged signals from white matter, cerebral spinal fluid and the global signal were regressed out. Finally, spatial smoothing was carried out with a 6 mm FWHM Gaussian kernel.

### Functional Parcellation and FCS Analysis of the Precuneus

The precuneus was defined based on the anatomical automatic label (AAL-90) template (Tzourio-Mazoyer et al., 2002). For each subject, an FC matrix was generated by performing the Pearson’s correlation (**r**) between the time series of each voxel and those of other voxels within the precuneus. This FC matrix was then subjected to Fisher’s Z transformation.

Next, the z-transformed FC matrix of each subject in the CN group was averaged to obtain a mean FC matrix. Functional parcellation (Kahnt et al., 2012; Kahnt and Tobler, 2017) was performed to this mean FC matrix using the K-means clustering algorithm (Tou and Gonzalez, 1974) with customized MATLAB (R2018a, The MathWorks Inc., Natick, MA, USA) scripts. By this means, voxels with similar intrinsic connectivity properties tend to be clustered together. *K* = 3 was specifically selected for this study based on consistent evidences demonstrating the tripartite anatomical and functional features of the precuneus (Margulies et al., 2009; Wang et al., 2019). Finally, the optimized three clusters were mapped to the precuneus of each subject.

To compute FCS, weak correlations less than 0.2 (Dai et al., 2015) that might arise from noises were set to zero (Dai et al., 2015). Then, the FCS for a given voxel **i** was calculated by averaging its FC to all other voxels (Liang et al., 2013):


$$\text{FCS }\left(i\right)\;=\;\frac{1}{n\;-\;1}\;\sum_{j\;=\;1,j\neq i}^{n}z_{ij,}r_{ij}\;>\;r_{0}$$

where **z_ij_** represents the z-value between voxel **i** and voxel **j**, and **n** represents the number of voxels in the precuneus.

### Statistical Analysis

All the statistical analyses were performed using the SPSS software (version 25.0, IBM Corporation, Armonk, NY, USA). Data normality was tested using the Kolmogorov-Smirnov test. Demographic and clinical characteristics were compared among the CN, MCI and AD groups using chi-square tests for categorical variables and one-way analysis of variance (ANOVA) with *post hoc* Bonferroni comparisons for continuous variables. For the whole precuneus and its subregions, the mean FCS and FDG-SUVR were obtained for each subject and compared among the three groups using analysis of covariance (ANCOVA) with *post hoc* least significant difference tests. Within each group, the mean FCS and FDG-SUVR were compared among the three subregions using repeated measures ANOVA with *post hoc* least significant difference tests. The three-sigma rule (three standard deviations) was applied to detect outliers. Multiple comparisons were corrected using Bonferroni corrections with an alpha threshold of 0.008 (i.e., 0.05/6, two imaging markers in three subregions) as statistically significant. Standardized z-scores of both FCS and FDG-SUVR were calculated so that they can be averaged across subjects. Spearman’s correlation analyses were then performed across voxels to explore the correlations between FCS and FDG-SUVR in the whole precuneus and its subregions. Finally, partial Pearson’s correlation analyses were performed to explore associations the FCS and FDG-SUVR of the whole precuneus and its subregions with MMSE scores in all three subject groups. Age, gender, and education level of each subject were controlled for the above ANCOVA and partial correlation analyses.

## Results

The demographic and clinical results of all participants are listed in Table 1. No significant difference was found in age, gender or education among the CN, MCI, and AD groups (*p* ≥ 0.183 for all occasions). Significantly decreased MMSE scores were found in AD patients compared to the MCI (*p* < 0.001) and CN (*p* < 0.001) groups, and in the MCI group compared to the CN group (*p* = 0.009).

**Figure 1:**
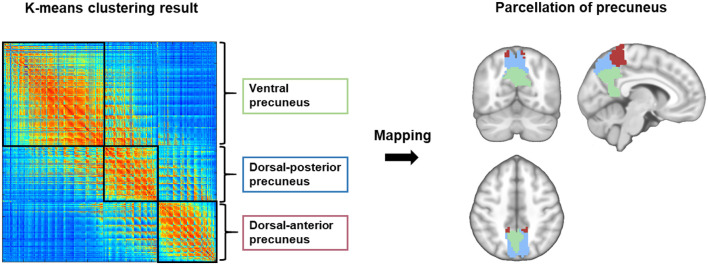
Functional parcellation of the precuneus. Using the K-means clustering algorithm, the precuneus was subdivided into the dorsal anterior (red), dorsal posterior (blue) and ventral (green) subregions based on its intra-regional functional connectivity.

Functional parcellation of the precuneus based on the K-means clustering algorithm is illustrated in Figure 1. The precuneus was functionally divided into the dorsal anterior (DA_pcu), dorsal posterior (DP_pcu), and ventral (V_pcu) subregions. In order to show the variation of functional subregions of the precuneus among different subjects, we performed functional parcellations for each CN subject and calculated the overlap ratios between each individual parcellation and the group parcellation results. On average, the overlap ratios (mean ± SD) for DA_pcu, DP_pcu, and V_pcu were 73% ± 11.1%, 61.2% ± 15.1%, and 70.9% ± 14.0%, respectively. Moreover, we performed functional parcellations for the MCI and AD groups to show that the subregions did not vary substantially across groups (see Supplementary Figure 1).

**Figure 2:**
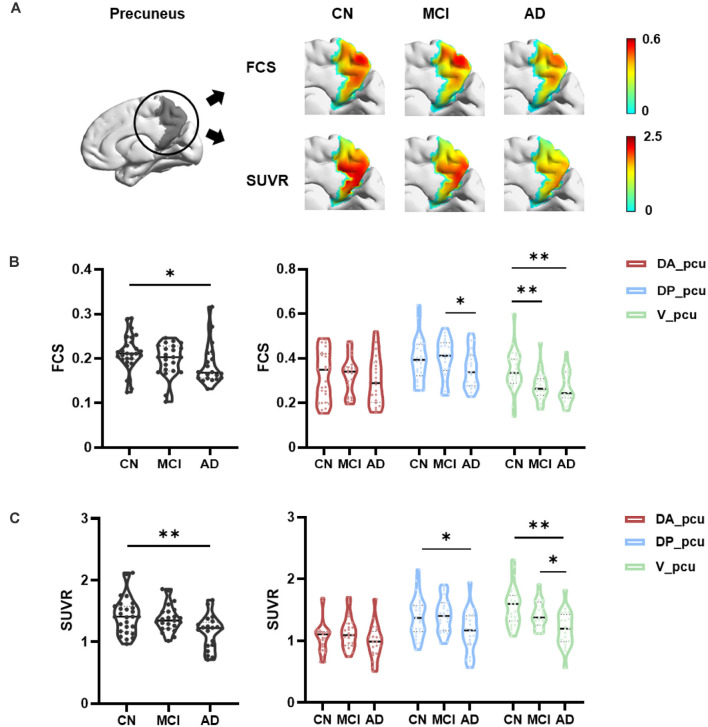
Differences in the FCS and SUVR in the whole precuneus and its subregions between the CN, MCI, and AD groups. **(A)** Maps of both FCS ad SUVR within the precuneus decreased in patients. **(B)** For the whole precuneus, decreased FCS was observed in the AD patients compared to the CN group. For the subregions of the precuneus, decreased FCS was found in DP_pcu in AD patients compared to the MCI group, and in V_pcu in patients with AD and MCI compared to the CN group. No difference was observed in the FCS of DA_pcu among the three groups. **(C)** For the whole precuneus, decreased SUVR was observed in AD patients compared to the CN groups. For the subregions of the precuneus, decreased SUVR was found in DP_pcu of AD patients compared to the CN group, and in V_pcu of AD patients compared to both the MCI and CN groups. No difference in the SUVR of DA_pcu was observed among the three groups. Between-group differences were compared *via* ANCOVA with *post hoc* least significant difference tests and Bonferroni corrections. Age, gender, and education were considered as covariates. *p < 0.05, uncorrected; **p < 0.05, Bonferroni corrected. Abbreviations: FSC, functional connectivity strength; SUVR, standard uptake value ratio; CN, cognitively normal; MCI, mild cognitive impairment; AD, Alzheimer’s Disease; V_pcu, ventral precuneus; DP_pcu, dorsal posterior precuneus; DA_pcu, dorsal anterior precuneus; ANCOVA, analysis of covariance.

Within the precuneus, maps of the FCS and FDG-SUVR both showed visually perceptible decreases in patients with MCI and AD, as shown in Figure 2A. The FCS of the whole precuneus and its subregions in the CN, MCI, and AD groups were displayed in Figure 2B. Specifically, the FCS of the whole precuneus showed a mild decrease in AD patients compared to CN subjects (*p* = 0.047). Looking into each subregion, we found that the FCS had significant decreases in V_pcu of AD patients (*p* = 0.008) and MCI patients (*p* = 0.006) compared to the CN subjects. Decreases in DP_pcu FCS were found in AD patients compared to the MCI (*p* = 0.011) and CN (*p* = 0.073, marginal difference) groups, and no changes were shown in the DA_pcu subregion. Glucose metabolism, estimated by FDG-SUVR, exhibited a similar pattern as FCS. As displayed in Figure 2C, significantly decreased SUVR of the entire precuneus was observed in the AD patients compared to the CN group (*p* = 0.006). In V_pcu, hypometabolism were found in the AD patients compared to both the CN (*p* < 0.001) and MCI (*p* = 0.045) groups, and in MCI patients compared to the CN group (*p* = 0.059, marginal difference). In DP_pcu, mild decreases were found in the AD patients compared to the CN subjects (*p* = 0.038) and MCI patients (*p* = 0.073, marginal difference). Finally, in DA_pcu, no significant changes were observed among the three subject groups. The quantitative values for both FCS and SUVR of all three groups are listed in Supplementary Table 1. Non-significant increases in the mean FCS and FDG-SUVR were observed in both DA_pcu and DP_pcu in the MCI group compared to the CN group.

**Figure 3:**
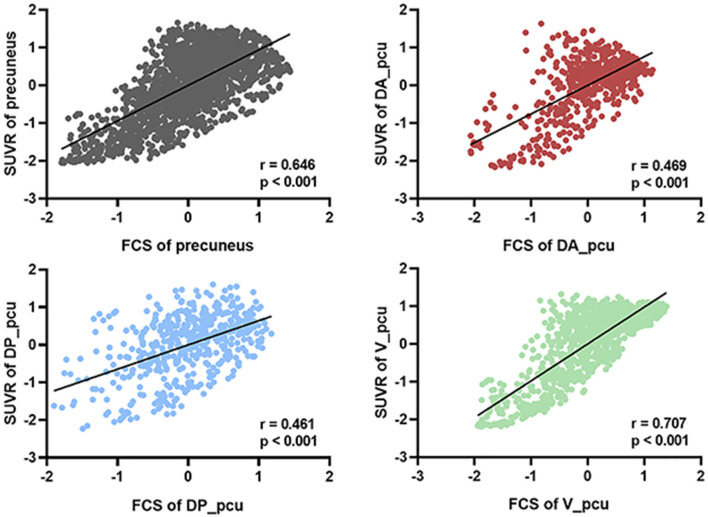
Voxel-based correlations between FCS and SUVR in the whole precuneus and its subregions. V_pcu (green) had the highest functional-metabolic correlation compared to the precuneus and other subregions. For each voxel, standardized z-scores of both FCS and SUVR were averaged across all subjects (*N* = 70). Spearmen’s correlation was performed between the two metrics across the voxels of the precuneus and each subregion. Abbreviations: FSC, functional connectivity strength; SUVR, standard uptake value ratio; V_pcu, ventral precuneus.

**Figure 4:**
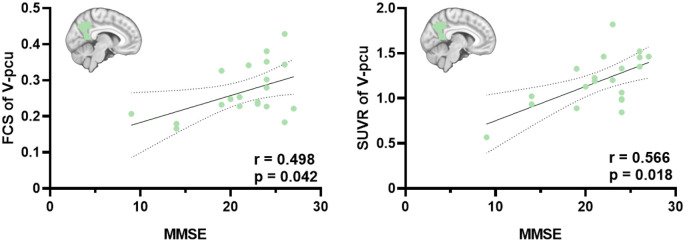
Significant correlations between FCS and MMSE scores and between SUVR and MMSE scores in the V_pcu of AD patients. Partial Pearson’s correlations were performed with age, gender and education were considered as covariates. Abbreviations: FSC, functional connectivity strength; SUVR, standard uptake value ratio; AD, Alzheimer’s Disease; V_pcu, ventral precuneus; MMSE, Mini-Mental State Examination.

We next investigated the associations between FCS and SUVR as well as between their reductions and cognitive decline in the whole and three subregions of the precuneus. As shown in Figure 3, V_pcu had the highest functional-metabolic correlation (*r* = 0.707, *p* < 0.001) within the precuneus. In addition, significant correlations between FCS and MMSE scores (*r* = 0.498, *p* = 0.042) and between SUVR and MMSE scores (*r* = 0.566, *p* = 0.018) were found in V_pcu only, as displayed in Figure 4 and Table 2. None of the other two subregions, or the whole precuneus, displayed significant correlations with cognitive declines.

**Table 2:** Associations of MMSE with FCS and SUVR in AD patients.

	Precuneus	DA_pcu	DP_pcu	V_pcu
FCS	*r* = 0.304	*r* = 0.338	*r* = 0.032	***r* = 0.498**
	*p* = 0.236	*p* = 0.185	*p* = 0.903	***p* = 0.042**
SUVR	*r* = 0.415	*r* = 0.056	*r* = 0.287	***r* = 0.566**
	*p* = 0.098	*p* = 0.831	*p* = 0.264	***p* = 0.018**

*Partial Pearson’s correlation analyses were performed with age, gender, and education as covariates. Abbreviations: FSC, functional connectivity strength; SUVR, standard uptake value ratio; AD, Alzheimer’s Disease; V_pcu, ventral precuneus; DP_pcu, dorsal posterior precuneus; DA_pcu, dorsal anterior precuneus; MMSE, Mini-Mental State Examination*.

**Figure 5:**
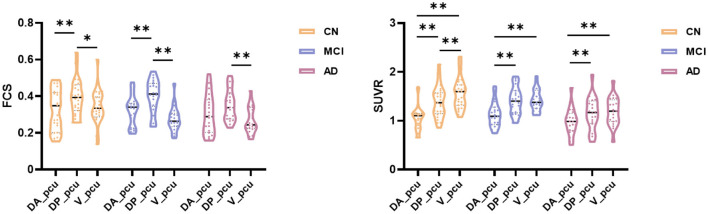
Subregional dedifferentiation of the FCS and SUVR in MCI and AD. For FCS, the significant difference between DP_pcu and DA_pcu that existed in CN and MCI groups disappeared in the AD group. For SUVR, the significant difference between DP_pcu and V_pcu that existed in the CN subjects decayed in both MCI and AD patients. Additionally, the overall analysis of variance (ANOVA) test yielded an insignificant main effect of subregion for the SUVR in the AD patients (*p* = 0.087). Subregional differences were compared *via* ANOVA with *post hoc* paired *t*-tests and Bonferroni corrections. *p < 0.05, uncorrected; **p < 0.05, Bonferroni corrected. Abbreviations: FSC, functional connectivity strength; SUVR, standard uptake value ratio; CN, cognitively normal; MCI, mild cognitive impairment; AD, Alzheimer’s Disease; V_pcu, ventral precuneus; DP_pcu, dorsal posterior precuneus; DA_pcu, dorsal anterior precuneus; ANOVA, analysis of variance.

As displayed in Figure 5, we observed that the significant difference in the FCS between DP_pcu and DA_pcu that existed in the CN and MCI groups disappeared in AD patients. Similarly are based on different methods of statistical analysis (i.e., ANCOVA with independent groups vs. ANOVA with repeated measures). In addition, Figure 2 reflects the characteristics of selective vulnerability of the subregions, whereas Figure 5 shows functional dedifferentiation within the precuneus in MCI and AD.

Finally, to investigate whether the results are dependent on the atlas used, we performed functional parcellation based on the precuneus defined by the Harvard-Oxford atlas (Desikan et al., 2006). As displayed in Supplementary Figure 2A, the precuneus was subdivided into four subregions, namely DA_pcu_HO, DP_pcu_HO, V_pcu_HO, and VP_pcu_HO. Among them, the first three subregions were similar as those defined by the AAL atlas. The additional VP_pcu_HO located at the posterior rim of the precuneus that was included by the HO atlas but not by the AAL atlas. Results of the statistical analysis are shown in Supplementary Figure 2B. The disruption patterns for both FCS and SUVR in DA_pcu_HO, DP_pcu_HO, and V_pcu_HO resemble those observed based on the AAL atlas. Specifically, V_pcu_HO was the earliest and most affected subregion. DP_pcu_HO was disrupted in AD patients whereas DA_pcu_HO did not exhibit significant changes. For VP_pcu_HO, a decrease in the SUVR in the AD group and no significant change in the FCS were observed. Overall, the functionally parcellated regions in our study could be consistently identified using different atlases, if the number of regions were appropriately selected. The main findings of the current study appear to be robust across different initial definitions of the precuneus.

## Discussion

In this study, a hybrid resting-state fMRI/FDG PET technique was employed to investigate the FCS and glucose metabolism in the precuneus and its subregions in groups of age- and education-matched healthy controls and patients with MCI and AD. Our results revealed a gradually disrupted pattern from the ventral precuneus in patients with MCI to dorsal precuneus in patients with AD. In particular, the ventral precuneus was the earliest affected subregion and its compromised FCS and glucose metabolism were associated with cognitive decline of AD patients.

The precuneus has been considered as a typical cortical hub region that not only is involved in complex and specialized cognitive functions but also plays a pivotal role in the inter-regional mediation of the brain. It is preferentially attacked by amyloid plaques (Buckner et al., 2009) and has drawn extensive attentions in AD-related studies. Previous evidences and this study (Figure 2) consistently found that the precuneus suffered disrupted functional integrity and glucose metabolism in patients with AD. Specifically, abnormal functional integrity measured by significantly reduced local and global FC (Greicius et al., 2004; Drzezga et al., 2011; Damoiseaux et al., 2012; Dai et al., 2015) as well as the amplitude of low-frequency fluctuations (ALFF) and regional homogeneity (ReHo) of the BOLD signal (He et al., 2007; Hafkemeijer et al., 2012; Liu et al., 2014; Marchitelli et al., 2018) has been observed in patients with AD compared to CN subjects. In addition, as a reflection of synaptic dysfunction, glucose hypometabolism in the precuneus of AD patients has been repetitively reported by FDG PET studies (Del Sole et al., 2008; Ye et al., 2020). In patients with MCI, decreased functional activity (e.g., FC between nodes of the DMN, ReHo and ALFF of the precuneus) was reported in most fMRI studies (Hafkemeijer et al., 2012; Xue et al., 2019). This study observed a non-significant decrease of FCS in MCI compared to CN groups, which may be due to the different biomarker (i.e., FCS) applied. The FCS represents the average FC between a given voxel and all voxels of the region of interest (Liang et al., 2013; Dai et al., 2015). The averaging process may decrease the sensitivity but increase the robustness in the evaluation of functional changes compared to other functional metrics. In addition, hypometabolism in the precuneus has been reported in MCI patients with increased risks of AD, which was suggested as a predictor for AD conversion (Mosconi, 2005; Mosconi et al., 2008; Bailly et al., 2015; He et al., 2015; Kato et al., 2016; Bauckneht et al., 2018; Ma et al., 2018). In our data, a nonsignificant decreasing pattern of the FDG-SUVR from CN to MCI was observed in Figure 2 and Supplementary Table 1. One possible reason behind this discrepancy may be a relatively older age (~70 years) of our MCI group (Kato et al., 2016.) suggested that more severe hypometabolism in the precuneus and PCC regions can be observed in early onset-AD (onset <65 years) compared to late onset AD (onset >65 years). Going along with our study, Nobili et al. (2008) did not find significant hypometabolism in the parietal cortex including precuneus in a group of MCI patients with a mean age of 75 years. Another reason may be attributed to the heterogeneity of the MCI population. Patients with MCI may exhibit different FDG PET patterns and develop different types of dementia. For example, the FDG PET pattern of frontotemporal dementia (FTD) does not include significant hypometabolism in the precuneus (Kato et al., 2016). In a group of 45 patients with MCI, Cerami et al., 2015) reported that 14 of them did not exhibit cortical hypometabolism, and six of them (five with behavioral variant of FTD-like and 1 with semantic variant of primary progressive aphasia-like patterns) did not show hypometabolism in the precuneus. We did not exclusively recruit MCI patients with the amnestic subtype or positive amyloid deposition, which may result in the nonsignificant hypometabolism in the precuneus. Therefore, explanations of the MCI-related findings in our study should be restricted to the aspects of cognitive impairment and not be extended to prodromal AD.

Apart from its clinical significance to AD as a whole brain structure, the precuneus has been widely recognized to be composed of anatomically and functionally heterogeneous subareas which could be differentially impacted by AD (Khan et al., 2020). Originated from its cytoarchitecture (Cavanna and Trimble, 2006) and agreed by studies utilizing anatomical (Wang et al., 2019) and functional connectivities (Margulies et al., 2009; Ye et al., 2021) in both humans and macaques, three distinct subregions have been suggested, namely the dorsal-anterior, dorsal-posterior and ventral precuneus. Specifically, whereas DA_pcu and DP_pcu are majorly associated with sensorimotor and visual functions, V_pcu is involved in higher-order cognitive functions such as memory and self-related processing. Recently, finer sub-parcellation results (i.e., 4–6 parcels) have been proposed (Cauda et al., 2010; Zhang and Li, 2012; Zhang et al., 2014; Fan et al., 2016; Luo et al., 2020), which made delicate subdivisions of the precuneus remain an open question. In several occasions, however, these finer subregions were hieratically categorized into the ordinary three clusters (Zhang and Li, 2012; Xia et al., 2014; Luo et al., 2020) when analyzing the functional heterogeneity. Therefore, we went along with the three-subregion functional parcellation scheme generated by a K-means clustering algorithm based on the FC within the precuneus (Zhang and Li, 2012).

One of our major findings is that V_pcu was the earliest and most affected subregion by AD, compared to the whole and other subareas of the precuneus. From a functional perspective (Figure 2A), it was the only subregion which showed significantly decreased FCS in MCI compared to normal controls. Moreover, the FCS within V_pcu demonstrated a more sensitive biomarker (with more stringent *p* values) as compared to the whole precuneus in patients with AD. To the best of our knowledge, disrupted FCS within V_pcu (or any subregions of the precuneus), as a reflection of local intrinsic FC, has not been previously reported. Disrupted global FC between V_pcu and other brain regions has been consistently shown by studies investigating differential functional architecture of the subregions of the posteromedial cortex (PMC) affected by AD (Xia et al., 2014; Wu et al., 2016; Khan et al., 2020). Among them, Wu et al. (2016) reported that disruptions began in a transitional region between the posterior cingulate cortex (PCC) and precuneus, which has an overlap with the V_pcu in this study, in mild AD patients and then spread to other subregions of PMC as the disease became more severe. Our study extended this finding by showing that the disruption in the FCS of V_pcu could occur in the MCI stage. This observation may be partially due to the fact that local FC has less variability across subjects than global FC (Tomasi and Volkow, 2011) and thus is more sensitive to between-group effects. In future studies, it will be very interesting to explore the FC of precuneus subdivisions with other brain functional networks. For example, since the ventral precuneus has a considerable overlap and stronger connectivity with the DMN (Cauda et al., 2010; Zhang and Li, 2012), we speculated that the within-network disturbances of V_pcu would also reflect on its FC with further regions of the DMN. A preliminary comparison using our current data showed a significant decrease in the FC between V_pcu and DMN in patients with MCI (*p* = 0.03) and a trend of decrease in patients with AD. Further explorations with a larger sample size are in merit to investigate this. From a metabolic perspective (Figure 2B), significantly decreased glucose metabolism was observed in the V_pcu of AD patients compared to both MCI patients and normal controls. The precuneus and its surrounding areas are among the regions that bear the highest metabolic rates in healthy subjects (Cavanna and Trimble, 2006) and suffer excessive glucose hypometabolism relative to atrophy in AD patients (Chételat et al., 2008; Karow et al., 2010). Our results further demonstrated that such AD-related hypometabolism was not homogeneous in the precuneus, with impairments mostly occurred in V_pcu. By exploring the relationship between FCS and SUVR in subregions of the precuneus, we observed that V_pcu had the highest functional-metabolic correlation (*r* = 0.707, *p* < 0.001) within the precuneus (see Figure 3), suggesting a tight association between decreased FC and lower neural activity in this subregion. Based on the connectivity degree-to-metabolism ratio, the ventral precuneus had even higher energy efficiency than cortical hubs (Tomasi et al., 2013). This connectivity-related high energy demand could render this subregion more vulnerable to Aβ deposition (Buckner et al., 2009; Drzezga et al., 2011; Khan et al., 2020) and might explain the sensitivity of V_pcu to neurodegeneration in patients with MCI and AD. In addition to the quantitative analysis applied in this study, metabolic connectivity (MC), a novel approach to measure interregional covariance of FDG PET (Lee et al., 2008; Shi et al., 2017, 2018), has received increasing attentions for differential diagnosis of dementing disorders (Shi et al., 2017; Titov et al., 2017). Specifically, reduced MCs in the precuneus and other regions have been reported in patients with prodromal AD (Morbelli et al., 2012), which has the potential to facilitate individual prediction of conversion from MCI to AD (Wang et al., 2020). Moreover, MC and FC may complement each other to reflect the coupling of energy utilization and neural synchronization in relative brain networks (Di and Biswal, 2012). In future work, MC can be applied to explore patterns of metabolic covariance in subregions of the precuneus and their disruptions with AD. Finally, the ventral precuneus participates in a spectrum of cognitive functions including self-reflective processing, such as self-awareness, and autobiographical/episodic memory retrieval, attention, language, emotion consciousness, etc. (Wang et al., 2019). As a result, disruptions in V_pcu were expected to be correlated with general cognitive decline in AD patients. Indeed, both decreased FCS and hypometabolism in V_pcu were better associated with the MMSE scores than the precuneus and its other subregions in AD patients (Figure 4 and Table 2). Together, our observations demonstrated that V_pcu had a higher sensitivity in the detection of MCI patients from the healthy subjects and contributed to the cognitive-related neurodegeneration in AD patients.

The dorsal portion of the precuneus was generally less impaired than the ventral portion in this cross-sectional study, revealing a gradually degenerative pattern from ventral to dorsal precuneus. In patients with MCI, we observed non-significant increases in both FCS and glucose metabolism in the dorsal subregions (Supplementary Table 1), which may account for the absence of difference against the CN group. In line with this, previous work also showed increased connectivity and metabolism in patients with MCI, which claimed that additional neural resources might be in need to compensate for neurodegeneration and maintain cognitive performance, supporting the compensatory-recruitment hypothesis (Nobili et al., 2008; Hafkemeijer et al., 2012; Kato et al., 2016; Xue et al., 2019). In patients with AD, the FCS and glucose metabolism slightly decreased in DP_pcu and maintained relatively intact in DA_pcu (Figure 2). The DP_pcu locates in a bridge area between V_pcu and DA_pcu and may play a transitional role in the spread of AD-related disruptions across the precuneus. Functionally, it strongly interacts with adjacent vision-related areas (Zhang and Li, 2012; Zhang et al., 2014) and connects to several components of the DMN (Xia et al., 2014). The DA_pcu, however, primarily connects to adjacent sensorimotor-related areas. Likely due to its positioning at the superior end of the progressive FC shifts in the precuneus (Cauda et al., 2010) and its less involvements in cognitive-related functions (Zhang and Li, 2012), the DA_pcu stayed rather functionally and metabolically conserved in AD patients. Going along with our findings, Marchitelli et al. (2018) reported that both glucose metabolism and ReHo decreased in a combined area of V_pcu and DP_pcu but not DA_pcu in a group of patients with AD or amnestic MCI compared to control subjects. For the global FC of the DP_pcu and DA_pcu with other brain regions, however, previous studies did not reach a consensus and reported both decreased or unchanged results (Xia et al., 2014; Wu et al., 2016; Khan et al., 2020). This discrepancy among existing evidences, in our opinion, reflects the expanding of progressive disruptions from ventral to dorsal precuneus in different cohorts of AD patients at a later stage. Additionally, we reported that differences in the FCS and glucose metabolism between DP_pcu and DA_pcu and between DP_pcu and V_pcu, respectively, which existed in CN subjects, diminished in patient groups (Figure 5). This observation indicated a dedifferentiation among the subregions of the precuneus in patients with MCI and AD.

This study has limitations. We recruited a modest sample size. In addition, there is a lack of information about the Aβ and tau status of the individuals or their precise protein pathology in the precuneus and its subregions. Regarding the MCI group, we did not explicitly include the amnestic subtype or evaluate their APOE status. Therefore, cautions should be used in relating the MCI-related results of this study to prodromal AD. Future studies are needed to validate our results with larger samples of patients with preclinical and prodromal AD.

In conclusion, this study evidenced heterogeneous susceptibilities of precuneus subregions to both functional and metabolic disruptions in patients with MCI and AD. A disruption pattern from ventral to dorsal precuneus, with V_pcu and DA_pcu being the most and least affected subregion, respectively, was shown.

## Data Availability Statement

Anonymized data are available upon collaborative requests. Further inquiries can be directed to the corresponding authors.

## Ethics Statement

The studies involving human participants were reviewed and approved by Institutional Review Board of Ruijin Hospital. The patients/participants provided their written informed consent to participate in this study.

## Author Contributions

YL, MZ, YZ, and BiaoL conceived and designed the study. MZ, BinyinL, GY, HM, XH, XL, JW, JL, and BiaoL recruited subjects and acquired the data. MZ, WS, ZG, JH, YZ, and YL analyzed the data and interpreted the results. YZ, WS, MZ, and YL drafted the manuscript. All authors contributed to the article and approved the submitted version.

## Conflict of Interest

The authors declare that the research was conducted in the absence of any commercial or financial relationships that could be construed as a potential conflict of interest.

## Publisher’s Note

All claims expressed in this article are solely those of the authors and do not necessarily represent those of their affiliated organizations, or those of the publisher, the editors and the reviewers. Any product that may be evaluated in this article, or claim that may be made by its manufacturer, is not guaranteed or endorsed by the publisher.
