# Supplementary material for: Simultaneous PET/fMRI Detects Distinctive Alterations in Functional Connectivity and Glucose Metabolism of Precuneus Subregions in Alzheimer’s Disease
Source: Front Aging Neurosci. 2021 Sep 24;13:737002. doi: 10.3389/fnagi.2021.737002 (PMC8498203; doi:10.3389/fnagi.2021.737002)
Supplement: Supplementary file 1 [file Data_Sheet_1.docx]

**Supplementary materials**


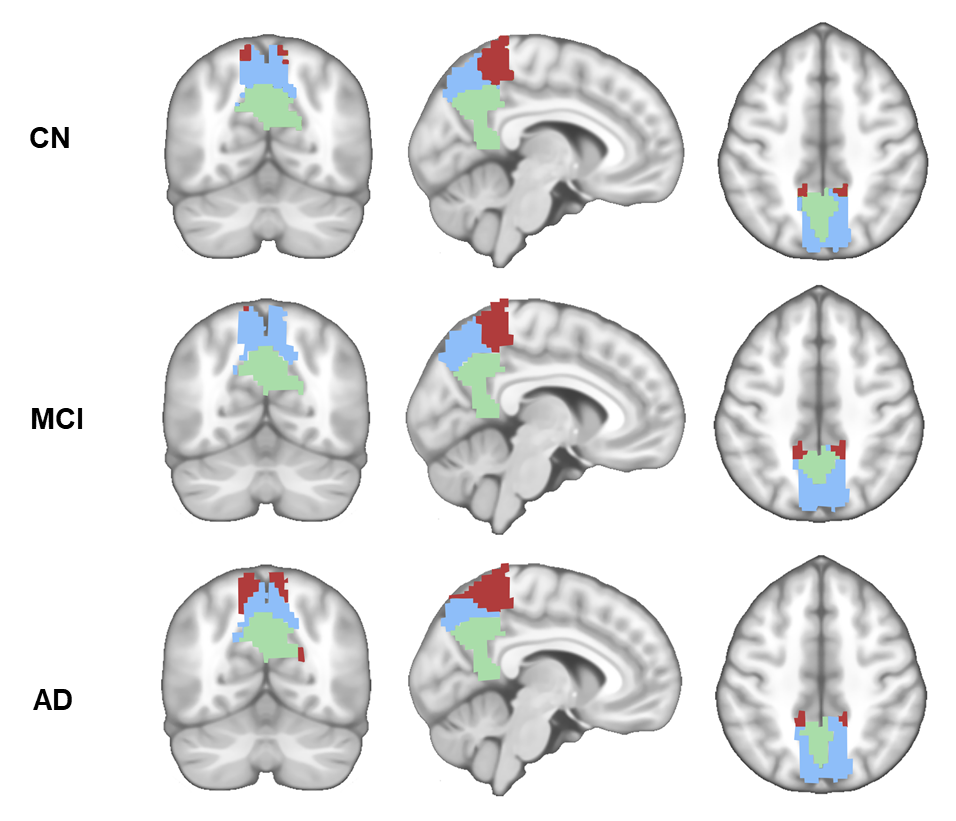


**Figure S1.** Functional parcellations of the CN, MCI, and AD groups.


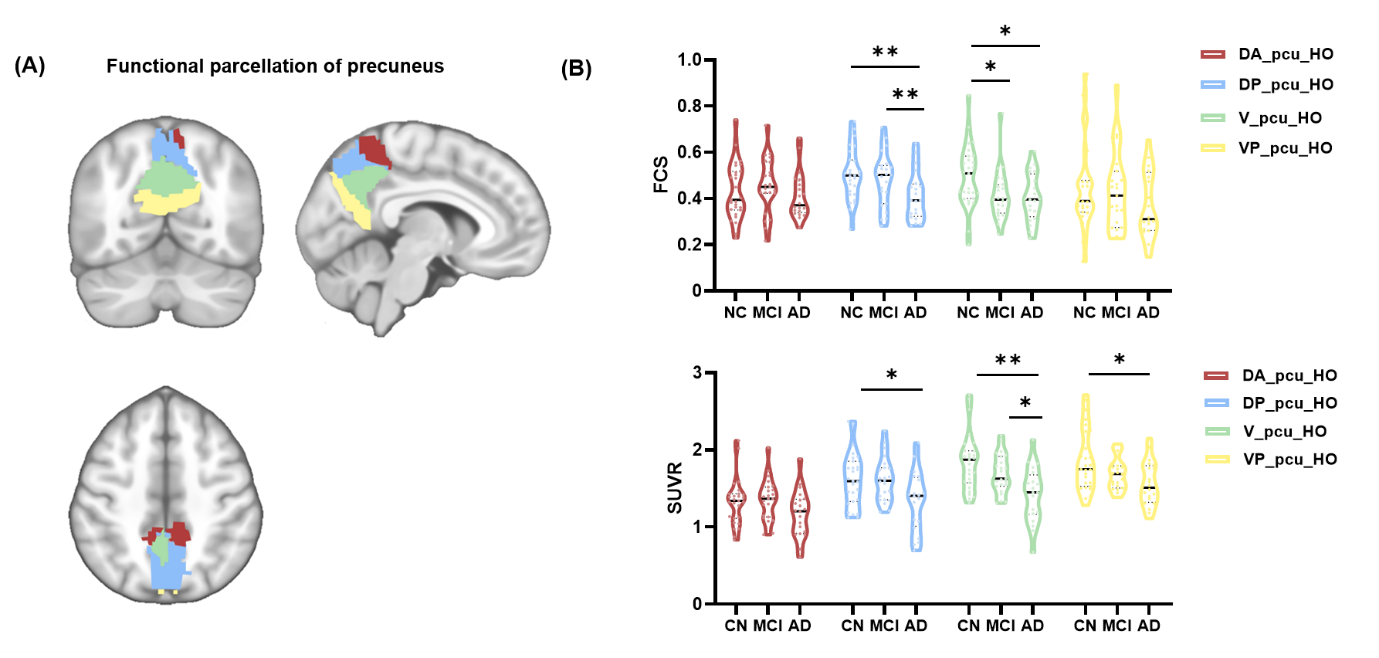


**Figure S2**. Validation of the results based on the Harvard-Oxford (HO) atlas. (A) The functional parcellation of the precuneus defined by the HO atlas (k = 4). The precuneus was subdivided into DA_pcu_HO, DP_pcu_HO, V_pcu_HO, and VP_pcu_HO. Among them, the first three subregions are similar as those defined by the AAL atlas. The additional VP_pcu_HO locates at the posterior rim of the precuneus that was included by the HO atlas but not by the AAL atlas. (B) Differences in the FCS and SUVR in the subregions between the CN, MCI, and AD groups. The disruption patterns for both FCS and SUVR in DA_pcu_HO, DP_pcu_HO, and V_pcu_HO resemble those observed based on the AAL atlas. Specifically, V_pcu_HO was the earliest and most affected subregion. DP_pcu_HO was disrupted in AD patients whereas DA_pcu_HO did not exhibit significant changes. For VP_pcu_HO, a decrease in the SUVR in the AD group and no significant changes in the FCS were observed. * p < 0.05, uncorrected; ** p < 0.05, Bonferroni corrected.

**Table S1**. Mean FCS and SUVR in the whole precuneus and its subregions in CN, MCI, and AD groups

|  |  | Precuneus | DA_pcu | DP_pcu | V_pcu |
| --- | --- | --- | --- | --- | --- |
| FCS | CN | 0.21 (0.04) | 0.32 (0.11) | 0.39 (0.10) | 0.34 (0.09) |
|  | MCI | 0.20 (0.04) | 0.34 (0.11) | 0.40 (0.09) | 0.28 (0.07) |
|  | AD | 0.19 (0.05) | 0.30 (0.11) | 0.35 (0.08) | 0.27 (0.07) |
| SUVR | CN | 1.42 (0.29) | 1.06 (0.24) | 1.39 (0.32) | 1.56 (0.32) |
|  | MCI | 1.38 (0.22) | 1.13 (0.25) | 1.41 (0.27) | 1.42 (0.22) |
|  | AD | 1.16 (0.27) | 0.99 (0.27) | 1.15 (0.34) | 1.19 (0.29) |

Values are mean (SD).

Abbreviations: FSC: functional connectivity strength; SUVR: standard uptake value ratio; CN: cognitively normal; MCI: mild cognitive impairment; AD: Alzheimer’s Disease; V_pcu: ventral precuneus; DP_pcu: dorsal posterior precuneus; DA_pcu: dorsal anterior precuneus.
